# Should a colon cancer screening decision aid include the option of no testing? A comparative trial of two decision aids

**DOI:** 10.1186/1472-6947-8-10

**Published:** 2008-03-05

**Authors:** Jennifer M Griffith, Marlie Fichter, Floyd J Fowler, Carmen Lewis, Michael P Pignone

**Affiliations:** 1 Center for Decision Making Research, Cecil Sheps Center for Health Services Research, University of North Carolina-Chapel Hill, Chapel Hill, North Carolina, USA; 2 Foundation for Informed Medical Decision Making, 40 Court Street, Suite 300, Boston, MA 02108, USA; 3 Division of General Internal Medicine, School of Medicine, University of North Carolina- Chapel Hill, Chapel Hill, North Carolina, USA

## Abstract

**Background:**

An important question in the development of decision aids about colon cancer (CRC) screening is whether to include an explicit discussion of the option of not being screened. We examined the effect of including or not including an explicit discussion of the option of deciding not to be screened in a CRC screening decision aid on subjective measures of decision aid content; interest in screening; and knowledge.

**Methods:**

Adults ages 50–85 were assigned to view one of two versions of the decision aid. The two versions differed only in the inclusion of video segments of two men, one of whom decided against being screened. Participants completed questionnaires before and after viewing the decision aid to compare subjective measures of content, screening interest and intent, and knowledge between groups. Likert response categories (5-point) were used for subjective measures of content (eg. clarity, balance in favor/against screening, and overall rating), and screening interest. Knowledge was measured with a three item index and individual questions. Higher scores indicated favorable responses for subjective measures, greater interest, and better knowledge. For the subjective balance, lower numbers were associated with the impression of the decision aid favoring CRC screening.

**Results:**

57 viewed the "with" version which included the two segments and 49 viewed the "without" version. After viewing, participants found the "without" version to have better subjective clarity about benefits of screening ("with" 3.4, "without" 4.1, *p *< 0.01), and to have greater clarity about downsides of screening ("with" 3.2, "without" 3.6, *p *= 0.03). The "with" version was considered to be less strongly balanced in favor of screening. ("with" 1.8, "without" 1.6, *p *= 0.05); but the "without" version received a better overall rating ("with" 3.5, "without" 3.8, *p *= 0.03). Groups did not differ in screening interest after viewing a decision aid or knowledge.

**Conclusion:**

A decision aid with the explicit discussion of the option of deciding not to be screened appears to increase the impression that the program was not as strongly in favor of screening, but decreases the impression of clarity and resulted in a lower overall rating. We did not observe clinically important or statistically significant differences in interest in screening or knowledge.

## Background

Colorectal cancer screening has been shown to be effective in reducing the incidence of, and mortality from, colorectal cancer [1]. Several different methods of screening are available, including fecal occult blood testing, sigmoidoscopy, combination of occult blood testing and sigmoidoscopy, colonoscopy, and possibly radiological tests such as barium enema and CT colography. The United States Preventive Services Task Force recommends colon cancer screening for adults age 50 to 75 [1]. Colon cancer screening was recently determined to be one of the highest priorities for preventive care in the US based on its high potential for reducing morbidity and mortality, and its cost-effectiveness [2].

Despite its potential efficacy, colon cancer screening remains underutilized, with approximately 50% of the age-eligible population up-to-date with screening in any form [3]. Several factors account for the low rates of utilization, including patient, provider and systems-related issues. Low levels of awareness among patients, and poor provider-patient communication are important barriers: most unscreened patients have never discussed screening with their providers [4-6]. Researchers have examined a number of potential means of addressing these barriers [7], including efforts to improve clinical decision making.

One potential tool for improving the quality of decision making and uptake of CRC screening is the use of a patient decision aid. Patient decision aids are tools developed to help patients make more informed, value-concordant decisions about health issues. They include information about the specific decision to be made, pros and cons of different alternatives, processes for identifying and clarifying values, and in some cases help with assuring that the patient's input is received and acted upon. Good decision aids are balanced, clear, and meet the needs of patients. Systematic reviews of decision aid studies have shown they can increase knowledge, reduce decisional conflict, and in some cases affect adoption of health behaviors [8-10].

Existing CRC decision aids [11-14] differ in the questions on which they focus: some have focused on the decision about which test to have done, assuming that some form of screening should be performed; others have addressed the question of whether or not to be screened [11,14]. An important question that arises in the development of decision aids about CRC screening is whether to include an explicit discussion of the option of not being screened as a viable alternative.

Proponents of including "no screening" as a viable option argue that the option of no screening should be included on the ethical grounds of fully promoting patient autonomy by allowing consideration of all possible alternatives [15,16]. Some also argue that CRC screening produces a relatively small reduction in the absolute risk of CRC-related mortality (1–2%) that, for some patients, may not outweigh the downsides of screening, including discomfort with the procedure, time lost from other activities, and cost. Including an explicit discussion of the option of "no screening" may also increase credibility of the decision aid [17-19] by providing a two-sided argument. Recent guidelines for decision aid developers have suggested that the option of no screening should be included, but do not comment on whether it should be presented only as a comparison to active choices or whether it should always be included as a viable option [20].

On the other hand, some argue that when evidence is felt to be strongly in favor of screening, as in the case of CRC screening, only active options should be explicitly discussed. The inclusion of a viable no screening option in a decision aid, especially when delivered by an expert, could confuse patients and result in no screening for patients who would otherwise choose to be screened. Others are concerned that despite the relatively small average reduction and potential for downsides of screening, CRC screening is relatively beneficial compared with other services and thus should be endorsed. With this line of reasoning, patients always have the option not to be screened, making it unnecessary to present it as a viable option.

Because both arguments, whether to include or not include an explicit discussion of not being screened, have merit and cannot be easily resolved, we sought to perform a comparative trial of two versions of a CRC screening decision aid. In this comparative trial we examine more closely the actual effects of including or not including the option of no screening as a viable choice. Our main outcomes were subjective measures of decision aid content, with secondary outcomes of screening interest and knowledge. Because the differences we were testing were values-based rather than knowledge-based, we expected that including or not including the option of no screening would mainly affect subjective measures of decision aid content, and possibly interest in screening, but were unlikely to affect knowledge.

## Methods

### Overview

Our study of including or not including a discussion of the option of no screening was part of a larger evaluation of a CRC screening decision aid sponsored by the Foundation for Informed Medical Decision Making (FIMDM), which also included a qualitative element not reported here. Our study used a targeted convenience sample to evaluate the two versions of the decision aid, one which included video segments with an explicit discussion of not being screened. Three study sites were included in the evaluation. Each site obtained Institutional Review Board approval for the study. Eligible patients completed informed consent at the beginning of their session. Study sites administered questionnaires prior to focus group discussions and provided resulting data to be aggregated for analyses and evaluation.

### Decision aid development and context of participant evaluation

The content of the CRC screening decision aid was based on a review of existing literature and was informed by previous decision aid development [13]. The development process began with a literature review of existing evidence and focus groups with patients and providers to identify the information to be included in the program. The evidence summary focused on information that a patient would need or should know to make an informed decision. The evidence summary was then integrated with results from the focus groups to produce the content document that served as the basis for decision aid production. A draft of the decision aid content then underwent a series of reviews by clinical and methodological experts. After this review, a "rough cut" of the decision aid was produced and used for the final evaluation by experts and patients. Our trial was completed as part of this final review process.

### Research question

To test the effect of providing the option of no screening as a viable choice, we produced two versions of the decision aid that differed only in the inclusion or exclusion of video segments in which two men (one a physician, the other a health services researcher) discussed their decisions about CRC screening. In one segment, one of the men discussed his decision against being screened, explaining that he felt the potential benefits were not worth the effort of screening; the other man discussed his decision to be screened to provide "peace of mind."

### The decision aid

Both versions of the decision aid were approximately 35 minutes in duration and were designed to educate patients and help them to make value-concordant decisions about CRC screening. Both versions first introduced the topics of colon cancer and screening. Introductory material also discussed lifetime colon cancer mortality risk with and without screening and how screening tests help detect colon cancer. The decision aid oriented viewers to the decision(s) they should consider while viewing the program; whether or not to have screening and, if screening is desired, which screening test to have. The next sections of the decision aid described the different types of colon cancer screening tests including: stool test for blood, sigmoidoscopy, a combination of stool test for blood and sigmoidoscopy, colonoscopy, and imaging tests (barium enema and CT colography). Each test was described in terms of how the test is completed, how often it needs to be completed, the amount of time needed to complete, effectiveness in finding polyps and cancer, convenience, discomfort, and risks associated with the test. The program concludes with the reminder that the individual first needs to decide whether or not to have screening. If they decide to have screening, they can then choose their preferred test.

Both versions of the decision aid include patients discussing their decisions about undergoing the different screening tests. In the version of the decision aid with the discussion of the option of deciding not to be screened ("with" version), the following segments are also included approximately 8 minutes into the program for initial statements and 25 minutes in for second statements.

### Endorsement of colon cancer screening; M. Barry, Physician

#### Initial statement

*I knew my risk of dying from colon cancer was small, but it was still on my mind and I reasoned that if I started a screening program I could lower that risk enough that I wouldn't have to worry about it anymore...and less things to worry about is a good thing in life from my perspective*.

#### Second statement

*A particular advantage of colon cancer screening for me is that by finding and removing polyps I can reduce my chance of getting colon cancer in the first place. Particularly with that advantage the risk and hassle is worth it for me*.

### Endorsement of no screening; D. Fryback, Health Services Researcher

#### Initial statement

*The cost of being screened for colon cancer and benefits are things that you have to think about. For me, the benefit's small. I think that there is not much chance of dying from colon cancer and reducing that small chance to a little smaller chance comes at a price*.

#### Second statement

*What are my chances of dying from colon cancer in my lifetime? The chance is very small, it is very, very small, and it doesn't go to zero if you get screened. What happens is that you get a small chance cut in half, if that, and that small change is just not worth it to me*.

These segments were not included in the "without" version of the decision aid. Both versions of the decision aid concluded with information designed to aid viewers in their decision process by having them think about their choices/options and what was right for them based on their values and beliefs. To assist viewers in the decision-making process, the program provided information comparing the different tests on aspects such as effectiveness and risks.

### Participants

**Figure 1:**
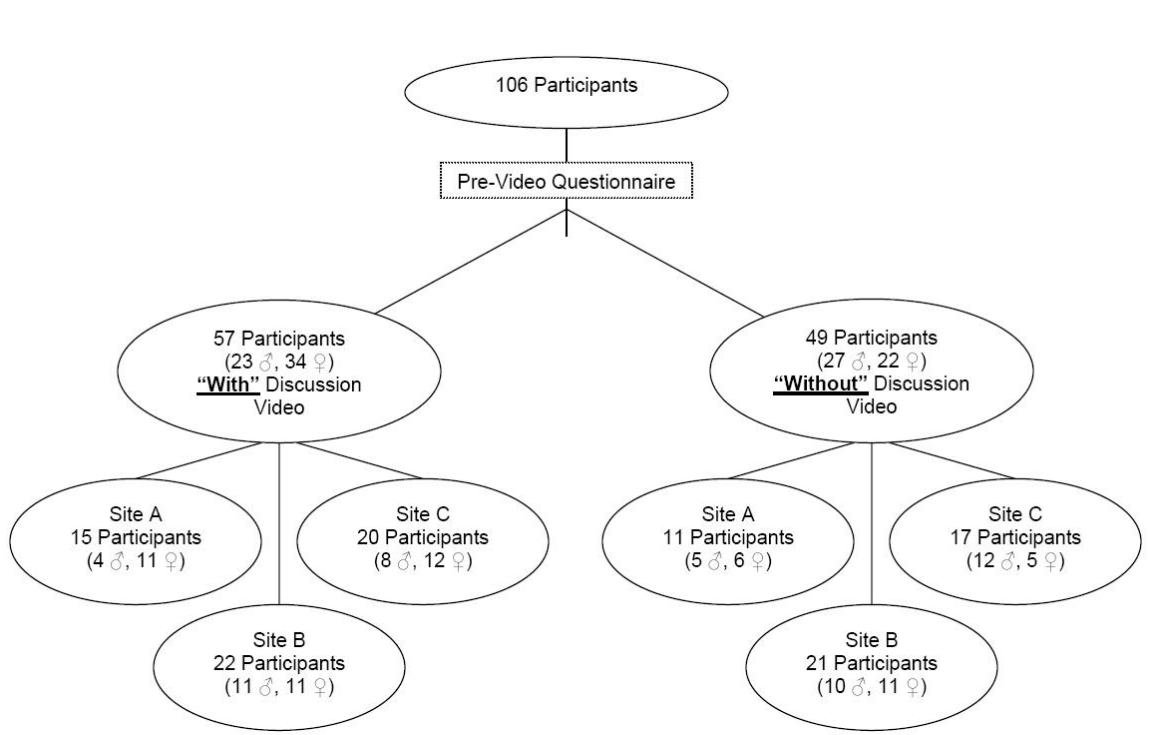
Study diagram.

Men and women ages 50–85 were recruited from three different communities (two in the Northeast and one in the Southeast of the United States). Sites were part of an existing collaborative effort sponsored by the FIMDM to evaluate decision aids. Recruiting technique varied by each site and included print advertising, telephone recruiting, and recruiting from a marketing database. Each site used a targeted convenience sample with the goal of recruiting approximately 12 participants to a group. Participants were assigned non-randomly to sex-specific groups that viewed either the decision aid version with the discussion of the no testing option or the version without this information (Figure 1). Non-random assignment was based on gender and the availability of the participant on scheduled focus groups dates. Men and women participated in different groups to remove the potential discomfort of discussing a sensitive subject with the opposite sex present [21].

### Measures

Our primary outcomes were subjective measures of decision aid content such as clarity and balance, and our secondary measures included knowledge and interest and intent to be screened. These measures were drawn from previous studies by our group, from previous evaluations used by the FIMDM, and a review of the key content of the decision aid (see Additional File 1 for questionnaires used in this study).

We assessed subjective measures of content after decision aid viewing, including the amount and clarity of information presented, and overall impression of the decision aid, using 5-point Likert response categories. The response categories used for amount of information were: 1- much less than I wanted, 2- a little less that I wanted, 3- about right, 4- a little more than I wanted, and 5- much more than I wanted. Lower scores were associated with the impression that the decision aid provided less information that wanted and higher scores with more information than was wanted. Other subjective measures, including clarity of information, ability to sort out what was important, ability to help make a decision, and overall impression of the decision aid, used response categories of: 1-Poor, 2- Fair, 3- Good, 4-Very Good, 5-Excellent. Higher numbers were associated with better subjective impressions of amount of information on advantages and disadvantages of screening, ability to sort out what was important, ability to help make a decision, and overall impression of the decision aid. For the subjective balance of the decision aid, lower numbers were associated with the impression of the decision aid favoring CRC screening and higher numbers against screening: Do you think the video was: 1- Strongly in favor of screening, 2- Somewhat in favor of screening, 3- Neither in favor of nor against screening, 4- Somewhat against screening, 5-Strongly against screening.

Interest and screening intent were assessed in all participants after viewing the decision aid, but were measured before decision aid viewing only for those who had not completed a colonoscopy within the last 10 years. Those who had completed other screening tests were able to respond to this question before viewing the decision aid because they could have chosen additional screening after learning about other options. The 5-point Likert response categories used for interest included: 1- Definitely not interested in being screened, 2- Probably not interested in being screened, 3- Not sure if I am interested or not, 4- Probably interested in being screened, 5- Definitely interested in being screened. The 4-point Likert response categories for intent were 1- Definitely don't intend to ask for screening, 2- Probably don't intend to ask for screening, 3- Probably intend to ask for screening, 4-Definitely intend to ask for screening. For both of these variables; higher scores were associated with greater interest in or intent for screening.

Participant knowledge was assessed before and after viewing the decision aid. Knowledge questions were developed based on the researchers' and developers' assessment of key content of the decision aid. We assessed knowledge to ensure that including or not including the discussion of no screening did not result in significant differences between the groups. The pre-decision aid knowledge index included three questions; the post decision aid knowledge index included these 3 questions and added two additional questions (five in total). These additional questions were not included in the pre decision aid questionnaire to keep participants from focusing on these specific points while viewing the decision aid. Scores for the 3-item index were computed with 1 point awarded for a correct response with a range of scores from 0 to 3. The two additional post-questionnaire knowledge questions were assessed separately.

We considered other measures, including decisional conflict, but decided against them to keep the questionnaire at a manageable length. Because we had identified a strong relationship between interest or intent to be screened after viewing and the subsequent completion of a CRC screening test in our earlier trial, we elected not to measure screening completion in this study [13].

### Statistical analyses

We used SAS software [22] and SPSS [23] for our analyses. Descriptive statistics were calculated for each variable. Due to skewed distributions, we used nonparametric Wilcoxon Rank Sum tests to compare subjective measures and screening interest and intent between groups after decision aid viewing. Wilcoxon Rank Sum tests were also used to compare pre knowledge scores between groups and post knowledge scores between groups. We used linear regression to determine whether age, sex, race, education level, screening status, or pre knowledge scores confounded the relationships of decision aid version with our outcome variables.

## Results

### Study population

Figure 1 shows the number of participants viewing each video. One hundred six adults participated in 12 total sessions at the three study sites. Each site conducted 4 focus groups sessions: two using the "with" version of the decision aid (1 male and 1 female session) and two using the "without" version of the decision aid (1 male and 1 female session).

Participants' mean age was 60, with a range of 50 to 81. A slight majority of participants were women (53%). Seventy six percent were white, and 22% were African American. Most participants (60%) had a 4-year college degree or more. Almost three quarters (72%) of participants were up-to-date with recommended colon cancer screening; 18% of participants had completed a home stool blood test (FOBT) within the last year, 19% completed sigmoidoscopy in the last five years, and 56% completed colonoscopy in the last ten years. There were moderate differences between groups in sex and being up-to-date with screening. We found no significant differences in pre-knowledge ("with" version 2.0, "without" version 2.1, *p *= 0.40) scores among those who viewed the "with" version compared with those viewing the "without" version (Table 1).

**Table 1:** Demographics by group

	"With" Version (with discussion of not screening) (n = 57)	"Without" Version (n = 49)	*p*-value
Mean Age	60 ± 7.0	60 ± 6.3	0.68
Sex- Female	60%	45%	0.17
Education Level*			0.77
Some college/2-year degree or less	40%	35%	
4-year college degree	21%	19%	
More than 4-year college degree	39%	46%	
Race			0.86
White	77%	71%	
Black/African American	19%	25%	
Up-To-Date with Screening	79%	63%	0.09
Pre-Knowledge Score^†^	2.0	2.1	0.40

* n = 48 for "without version"

† Knowledge scores computed with 1 for correct 0 for incorrect and not answered; range 0–3, Wilcoxon Rank Sum test statistic

### Subjective impressions of amount, clarity, and balance of information

Table 2 shows that the "without" version was found to have better clarity of information on benefits, clarity of information on downsides, amount of information on downsides of screening, and helping patients prepare for making a decision. For both versions, 16% of participants indicated that the decision aid was "balanced"; however, most indicated that it was either strongly or somewhat in favor of screening. The "with" version, which included the discussion of the no screening option, was scored as being less strongly in favor of screening, but scored lower on overall impression than the "without" version. The two versions did not differ in other subjective measures, including amount of information on benefits and sorting out what was important.

**Table 2:** Subjective ratings of videos by group

	**"With" Version **(with discussion of not screening) Mean n = 57	**"Without" Version** Mean n = 49	***p*-value**
Clarity of information on benefits*	3.4	4.0	<0.01
Amount of information on benefits*	2.9	2.9	0.72
Clarity of information on downsides	3.2	3.6	0.03
Amount of information on downsides	2.7	3.0	0.02
Helped me sort what was important	3.5	3.8	0.13
Helped me prepare for making a decision^†^	3.5	3.9	0.03
Balance^†^	1.8	1.6	0.05
Overall rating of video^†^	3.5	3.8	0.03

Measured with 5 point Likert response categories, Wilcoxon Rank Sum test statistic

† n = 56 for "with" version

* n = 48 for "without" version

### Differences in knowledge between groups

We found no significant differences in post-knowledge ("with" version 2.3, "without" version 2.3, *p *= 0.72) scores among those who viewed the "with" version compared with those viewing the "without" version. Change scores did not differ significantly either ("with" version +0.37, "without" version +0.24, *p *= 0.35). We also did not find any significant difference between the groups on the two additional knowledge questions included on the post-questionnaire. Eighty-seven percent viewing the "with" version and 86% of those viewing the "without" version correctly answered that most polyps will not develop into cancer if not removed (*p *= 0.78). Ninety-seven percent viewing the "with" version and 98% viewing the "without" version correctly responded that less than 10 out of 100 adults would develop colon cancer over their lifetime (*p *= 1.00).

### Interest and intent for screening post decision aid between groups

No significant differences were identified in interest and intent among all participants responding after viewing the decision aid (Table 3), or in the sub-group of participants not up-to-date with screening (data not shown).

**Table 3:** Screening interest and intent by group all participants responding post video

	**"With" Version **(with discussion of not screening) Mean	**"Without" Version** Mean	***p-value***
Interest in screening^†^	3.4	3.5	0.54
Intent to be screened*^‡^	2.9	2.8	0.65

† n = 53 for "with" version and n = 45 for "without" version

* n = 50 for "with" version and n = 46 for "without" version

‡Measured on a 4-point Likert Scale, Wilcoxon Rank Sum test statistic

### Multivariate analyses

Relationships between version and subjective measures, interest, or knowledge, did not change when we controlled for age, sex, race, education level, screening status, or pre-knowledge scores in multiple linear regression.

## Discussion

In a comparative trial, we evaluated two versions of a decision aid that differed only in the inclusion or exclusion of two segments, one of which included an explicit discussion and endorsement of the option of deciding not to be screened. The version with the explicit discussion of the option of no screening ("with" version) was rated subjectively as less strongly in favor of CRC screening, but had lower subjective rating of clarity and a lower overall rating. We found no statistically significant or clinically important differences between versions in terms of interest or intent for screening, or knowledge.

We are unaware of other studies that have directly compared including or not including information describing the option of "no screening" within the same decision aid. Two previous CRC decision aid studies included information about the option of not being screened and compared it against a minimal information control group. Both studies were small, but did not find differences in screening between groups [11,14]. The decision aid by Wolf and colleagues included this explicit statement that "another option is to not be tested at all, unless signs of colon cancer develop, though at that point, cancer is less likely to be curable" [14]. Dolan and colleagues used an analytical hierarchy model for "choosing [the] best approach for colorectal cancer screening" which included a "wait & see" option that described the option of not being screened [11]. Previous iterations of a CRC decision aid developed by our team have not included the explicit option of "no screening" and have shown increases in interest and intent to be screened, as well as actual increases in screening rates [13].

A number of questions arise when determining the content of decision aids including the amount and type of information that they should attempt to communicate. In another lab-based study, we have explored the amount of screening information provided, namely the number of screening tests discussed in a decision aid [24]. This work also found that the number of tests discussed in a decision aid did not affect interest in screening.

Another question to consider is who communicates the information in a decision aid. The "with" version of the decision aid included an endorsement of the option of no screening by a health services researcher and to balance this, a physician endorsing the option to be screened. Opposing endorsements may have contributed to the perception of balance in this version but also may have reduced clarity. Although we did not test the specific effect of expert versus non-expert endorsements, nor the type of expert providing the endorsement, the subjective differences noted between the two versions may be affected by endorsements and who provides them.

In this case, the endorser of the option of no screening placed considerable value on the time requirements for screening and made a judgment about the chance of a potential benefit of screening relative to this time cost. If the viewer holds a different value for his or her time, or feels differently about the chance of benefit, he or she may be misled by the endorser's conclusion. Neutral representation of the options, both by a narrator or even graphical representations, may produce different effects, and should be compared in a future study [25]. Testing the effect of vignettes versus other methods is a high priority for research [26].

Our study has several limitations. First, it is a small study, which does not allow us to definitively rule out small but meaningful differences between groups. We examined several subjective outcomes, and did not adjust for multiple comparisons, so our findings should be interpreted cautiously. Second, the knowledge questions that we used did not evaluate participant's knowledge of the relative risks and benefits of the various screening options. Hence, they may not be ideal for evaluating whether the decision aid fulfilled the goal of educating patients about the relative risks and benefits of the various screening options available. Third, we did not measure actual screening behavior, so it is possible that the two versions would have different effects on that endpoint. Our previous studies have found strong correlations between interest or intent and actual test completion suggesting that those who indicate interest and intent are more likely to complete screening [13]. Nevertheless, our findings should be confirmed in a larger randomized trial of unscreened individuals viewing the decision aid that also measures whether screening is completed. In addition, we did not assign participants randomly to the two study groups, instead allocating by gender and participant availability. We did note some differences between groups at baseline, but controlling for them did not affect our findings. We had diverse geographic representation, but our participants were volunteers, the majority had undergone some form of screening, and were highly educated, affecting the finding's generalizability to other clinical populations.

It is also important to note that the amount of the decision aid devoted to the discussion of "no screening" was less than two minutes in a 35 minute program, and that overall both programs were seen as favoring screening. The differences between the decision aids were mainly related to the effect of how the health services researcher and physician interviewed with opposing views on screening valued the magnitude of benefit from screening and did not differ in terms of factual information on risks and benefits. It is possible that our results would differ if a greater proportion of time was spent on discussion of the option of "no screening."

Our study examines the question of colorectal cancer screening, where the evidence of benefit is relatively strong and its magnitude of effect relatively large. These results may not generalize to other decisions where the evidence is more uncertain, such as prostate cancer screening, mammography for women in their forties, or decisions about heart disease prevention.

## Conclusion

We believe our study provides important information for researchers and developers of decision aids and those who seek to increase their use in clinical practice. Including an explicit discussion of the option of "no screening" appears to increase the impression of "balance" away from strongly favoring screening but decreases the impression of clarity and overall rating, without large effects on screening interest, intentions, or knowledge. Further research is required to better understand the optimal content of decision aids that will promote better quality decision making.

## Abbreviations

CRC – colorectal cancer; FIMDM – Foundation for Informed Medical Decision Making

## Competing interests

This study was funded by the Foundation for Informed Medical Decision Making. The Foundation is a not-for-profit organization that creates decision support materials and funds research about how help best to help patients making decisions. Ms Fichter and Dr. Fowler are both employed by the Foundation.

## Authors' contributions

JMG drafted and revised the manuscript. MF, FJF, MPP conceived of the study, and participated in its design and coordination, statistical analyses, and drafted and revised the manuscript. CLL helped draft and revise the manuscript and interpret results. All authors read and approved the final manuscript.

## Pre-publication history

The pre-publication history for this paper can be accessed here:



## Supplementary Material

Additional File 1 Study Questionnaires. The pre and post decision aid questionnaires used in the study are provided. Click here for file
