# Supplementary material for: Should a colon cancer screening decision aid include the option of no testing? A comparative trial of two decision aids
Source: BMC Med Inform Decis Mak. 2008 Mar 5;8:10. doi: 10.1186/1472-6947-8-10 (PMC2275224; doi:10.1186/1472-6947-8-10)
Supplement: Additional File 1 — Study Questionnaires. The pre and post decision aid questionnaires used in the study are provided. [file 1472-6947-8-10-S1.doc]

**Colon Cancer Screening Video Pre-Test**

*Please mark the best answer to the questions below.*

1. Have you completed a home stool blood test?

[ ] Yes, within the last year

[ ] Yes, but not within the last year

[ ] No

1. Have you had a sigmoidoscopy?

[ ] Yes, within the last 5 years

[ ] Yes, but not within the last 5 years

[ ] No

1. Has your doctor given you a rectal exam in the last 2 years?

[ ] Yes

[ ] No

1. Have you completed a colonoscopy?

[ ] Yes, within the last 10 years (skip to #8)

[ ] Yes, but not within the last 10 years (skip to #6)

[ ] No

1. Have you ever discussed colon cancer screening with your doctor?

[ ] Yes

[ ] No

1. Are you interested in being screened for colon cancer in the next 6 months? (check one)

[ ] Definitely interested in being screened

[ ] Probably interested in being screened

[ ] Not sure if I am interested or not

[ ] Probably not interested in being screened

[ ] Definitely not interested in being screened

1. Do you intend to ask your doctor about being screened for colon cancer in the next 6 months? (check one)

[ ] Definitely intend to ask for screening

[ ] Probably intend to ask for screening

[ ] Probably don’t intend to ask for screening

[ ] Definitely don’t intend to ask for screening

1. At what age do you think people at average risk who want to be screened should start getting regular screening tests for colon cancer? (check one)

[ ] 35

[ ] 40

[ ] 50

[ ] 55

1. Which of the following tests is **not** recommended to be used alone as a screening test for colon cancer? (check one)

[ ] Stool blood test (or fecal occult blood test)

[ ] Sigmoidoscopy

[ ] Colonoscopy

[ ] Digital rectal exam

1. There is no value in getting tested for colon cancer if you do not have any symptoms.

[ ] True

[ ] False

1. How old were you on your last birthday? ______ years
2. Are you:

[ ] Male

[ ] Female

1. What is the highest grade or level of school that you have completed? (check one)

[ ] 8^th^ grade or less

[ ] Some high school, but did not graduate

[ ] High school graduate or GED

[ ] Some college or 2-year degree

[ ] 4-year college graduate

[ ] More than 4-year college degree

1. How would you describe yourself?

[ ] American Indian or Alaskan Native

[ ] Asian or Pacific Islander

[ ] Black or African-American

[ ] Latino

[ ] White

[ ] Another race or multiracial (write in) ____________________

**Thank You.**

**Colon Cancer Screening Post-Test**

*Please mark the best answer to the questions below.*

1. The makers of this video want to show why patients, and not just doctors, should participate in deciding about colon cancer testing. How would you rate the video’s ability to do this? (check one)

[ ] Poor

[ ] Fair

[ ] Good

[ ] Very good

[ ] Excellent

1. How would you rate the **length** of the video? (check one)

[ ] Should be much shorter

[ ] Should be a little shorter

[ ] About right

[ ] Could be a little longer

[ ] Could be much longer

1. The **amount** of information about the possible **benefits** of colon cancer screening was: (check one)

[ ] Much less than I wanted

[ ] A little less than I wanted

[ ] About right

[ ] A little more than I wanted

[ ] Much more than I wanted

1. The **clarity** of the information about the possible **benefits of colon cancer screening** was: (check one)

[ ] Poor

[ ] Fair

[ ] Good

[ ] Very good

[ ] Excellent

1. The **amount** of information about the possible **disadvantages** of colon cancer screening was: (check one)

[ ] Much less than I wanted

[ ] A little less than I wanted

[ ] About right

[ ] A little more than I wanted

[ ] Much more than I wanted

1. The **clarity** of the information about the possible **disadvantages** of colon cancer screening was: (check one)

[ ] Poor

[ ] Fair

[ ] Good

[ ] Very good

[ ] Excellent

1. How would you rate this video’s ability to help people facing this decision **sort out what is important to them**? (check one)

[ ] Poor

[ ] Fair

[ ] Good

[ ] Very good

[ ] Excellent

1. A goal of this video is to help people **prepare to talk with their doctors** about colon cancer screening. How would you rate the video’s ability to do this? (check one)

[ ] Poor

[ ] Fair

[ ] Good

[ ] Very good

[ ] Excellent

1. Another goal of this video is to help people **prepare to make a decision** about colon cancer screening. How would you rate the video’s ability to do this? (check one)

[ ] Poor

[ ] Fair

[ ] Good

[ ] Very good

[ ] Excellent

1. How would you rate the video’s **ability to hold your interest**? (check one)

[ ] Poor

[ ] Fair

[ ] Good

[ ] Very good

[ ] Excellent

1. **Overall**, how would you rate the video? (check one)

[ ] Poor

[ ] Fair

[ ] Good

[ ] Very good

[ ] Excellent

1. Do you think the video was: (check one)

[ ] Strongly in favor of screening

[ ] Somewhat in favor of screening

[ ] Neither in favor of nor against screening

[ ] Somewhat against screening

[ ] Strongly against screening

1. In terms of the different ways of being screened, do you think the video: (check one)

[ ] Favored FOBT (or Fecal Occult Blood Test)

[ ] Favored sigmoidoscopy

[ ] Favored colonoscopy

[ ] Favored radiological screening (barium enema or CT)

[ ] Did not favor any one approach over the others

1. Are you interested in being screened for colon cancer in the next 6 months? (check one)

[ ] Definitely interest in being screened

[ ] Probably interested in being screened

[ ] Not sure if I am interested or not

[ ] Probably not interested in being screened

[ ] Definitely not interested in being screened

1. Do you intend to ask your doctor about being screened for colon cancer in the next 6 months? (check one)

[ ] Definitely intend to ask for screening

[ ] Probably intend to ask for screening

[ ] Probably don’t intend to ask for screening

[ ] Definitely don’t intend to ask for screening

1. If you were going to be tested, which test would you want to have? (check one)

[ ] FOBT (Fecal Occult Blood Test) each year alone

[ ] Sigmoidoscopy every 5 years alone

[ ] FOBT each year and sigmoidoscopy every 5 years

[ ] Colonoscopy every 10 years

[ ] Barium enema or CT colography every 5 years

1. How confident are you that this is the best decision for you? (check one)

[ ] Not at all confident

[ ] A little confident

[ ] Somewhat confident

[ ] Very confident

1. At what age should people at average risk who want to be screened start getting regular screening tests for colon cancer? (check one)

[ ] 35

[ ] 40

[ ] 50

[ ] 55

1. Which of the following tests **not** recommended to be used alone as a screening test for colon cancer? (check one)

[ ] Stool Blood Test (or fecal occult blood test)

[ ] Sigmoidoscopy

[ ] Colonoscopy

[ ] Digital Rectal Exam

1. About how many polyps will develop into cancer if not removed? (check one)

[ ] Most will

[ ] About half will

[ ] Most will not

1. There is no value in getting tested for colon cancer if you do not have any symptoms.

[ ] True

[ ] False

1. Out of 100 adults, about how many will develop colon cancer in their lifetime? (check one)

[ ] Fewer than 10

[ ] 10-19

[ ] 20-29

[ ] 30 or more

**Thank You.**
